# Electrospun Polyvinylpyrrolidone Fibers for Fast-Dissolving Drug Delivery: Defining the Viscosity Window and Evaluating the Role of Molecular Weight

**DOI:** 10.3390/pharmaceutics18091056

**Published:** 2026-08-25

**Authors:** Luca Éva Uhljar, Zsófia Viktória Tagscherer, Rita Ambrus

**Affiliations:** Institute of Pharmaceutical Technology and Regulatory Affairs, Faculty of Pharmacy, University of Szeged, Eötvös Street 6, H-6720 Szeged, Hungary; uhljar.luca.eva@szte.hu (L.É.U.);

**Keywords:** disintegration, drug delivery system, electrospinning, fast-dissolving, microfiber, nanofiber, orodispersible, polyvinylpyrrolidone, PVP, viscosity

## Abstract

**Background/Objectives**: Electrospun polyvinylpyrrolidone (PVP) fibers are highly promising for fast-dissolving drug delivery. **Methods**: In this study, five PVP grades with molecular weights ranging from 4000 to 1,300,000 Da were investigated over a broad concentration range (10–60 *w/w*%) to evaluate their electrospinnability and dissolution behavior. **Results**: A well-defined viscosity window of approximately 150–680 mPa·s was identified for the formation of continuous, bead-free fibers. Deviations from this optimal window resulted in electrospraying or jet instability. Continuous fibers were successfully prepared from all investigated PVP grades, including low-molecular-weight PVP K-12 (Mw 4000), demonstrating that appropriate solution viscoelasticity can compensate for limited chain entanglement. Remarkably, in vitro testing revealed that all fibrous formulations exhibited ultrafast disintegration (0.29–1.33 s) and dissolution (0.39–2.32 s). Statistical analysis confirmed no significant differences attributable to polymer molecular weight or fiber diameter, effectively challenging the common assumption that higher-molecular-weight PVP delays disintegration. Instead, the immediate dissolution originates from rapid wetting and capillary-driven fluid uptake, facilitated by the highly porous nano- and microfibrous network. **Conclusions**: By highlighting the dominant role of macroscopic structural properties over polymer chain length, these findings provide a practical framework for the development of fast-dissolving electrospun PVP-based drug delivery systems.

## 1. Introduction

**Figure 1:**
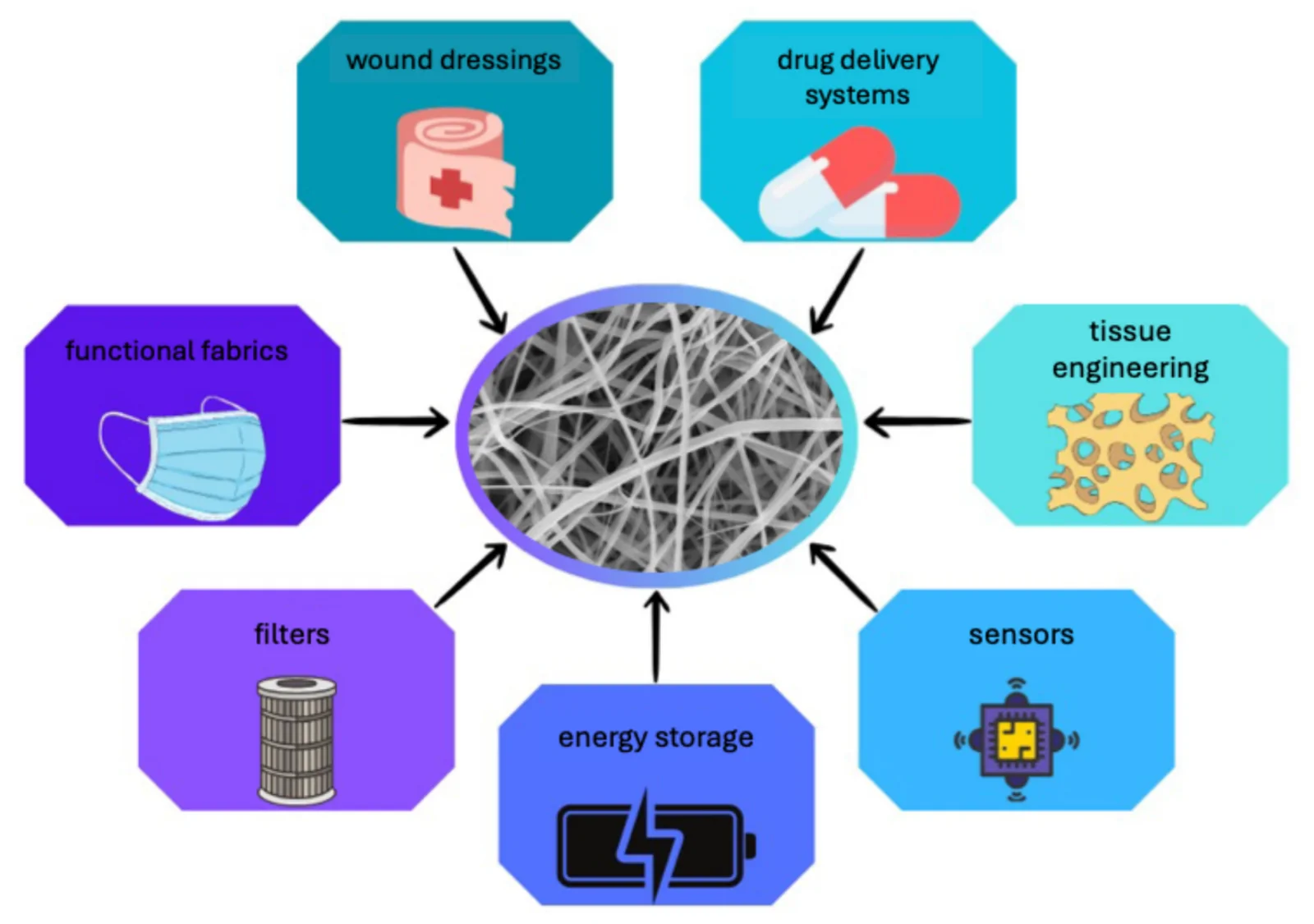
Major application fields of nanofibrous materials.

Electrospun nanofibrous membranes have attracted increasing attention in recent years due to their unique structural properties, including high specific surface area, tunable porosity, and favorable mechanical characteristics. These features make them highly versatile materials for a wide range of industrial and scientific applications (Figure 1).

In environmental engineering, they are widely used in air and water filtration systems for the efficient removal of fine particulate matter, microorganisms, and heavy metal ions [1,2]. In the field of energy storage, electrospun nanofibers serve as separators in lithium-ion batteries and as electrode materials in supercapacitors, contributing to improved electrochemical performance [3,4]. In sensor technology, electrospun nanofibrous materials are widely employed as active components in chemiresistive gas sensors owing to their high sensitivity, rapid response–recovery dynamics, and enhanced gas adsorption capability [5]. In the textile industry, electrospun nanofibers enable the fabrication of functional fabrics with properties such as antibacterial activity, water repellency, and enhanced breathability, particularly for protective clothing [6]. In the biomedical field, they are extensively investigated as scaffolds for tissue engineering, wound dressing materials, and drug delivery systems owing to their ability to mimic the extracellular matrix and support controlled release of therapeutic agents [7,8].

These examples demonstrate the broad scientific relevance of nanofibers and their extensive investigation across diverse research fields. Within this rapidly expanding area, the present work focuses on the development of drug-loaded nanofibers intended for pharmaceutical applications.

Poly(vinylpyrrolidone) (PVP) is a water-soluble synthetic polymer produced by the polymerization of N-vinylpyrrolidone monomers. It is available in a wide range of molecular weights and is extensively utilized due to its inertness, non-toxicity, thermal stability, pH stability, and excellent biocompatibility [1]. Owing to its favorable properties and ease of processing, PVP has become a commonly used material for the fabrication of nanofibers as drug delivery systems [2,3,4].

Electrospinning is the most widely used method for nanofiber production due to its simplicity and scalability. In nozzle-based electrospinning, a polymer solution is delivered through a needle at a controlled flow rate and subjected to a high electric field, which induces the formation of a fine jet ejected from the needle tip. As the jet travels toward a grounded collector, it undergoes continuous stretching and solvent evaporation, resulting in the formation of nanofibers that are deposited as a fibrous membrane [5].

The parameters of the electrospinning significantly influence the properties of the resulting nanofibers. These can be categorized into three key groups: (i) process parameters, including applied voltage, flow rate, and tip-to-collector distance; (ii) material properties, such as solution viscosity, surface tension, and electrical conductivity; and (iii) environmental conditions, including temperature and relative humidity [6]. As these parameters are strongly interdependent, their optimization is essential for achieving nanofibers with controlled morphology and diameter.

Among material parameters, polymer molecular weight and concentration play a fundamental role in determining electrospinning behavior, primarily through their influence on solution viscosity and the degree of chain entanglement. Higher-molecular-weight polymers generally promote bead-free fiber formation and tend to produce fibers with larger diameters [7]. In cases where high-molecular-weight polymers are unavailable, increasing solution concentration can provide sufficient chain entanglement. Indeed, polymer concentration critically determines fiber morphology. Insufficient concentrations lead to bead formation due to inadequate chain overlap, whereas excessively high concentrations lead to the formation of discontinuous fibers or induce premature solvent evaporation at the needle tip, resulting in clogging and defective fibers [8]. Thus, solution viscosity is a critical determinant of electrospinning performance and is strongly influenced by both the molecular weight of the polymer and the concentration of the solution.

In pharmaceutical technology, the disintegration behavior of nanofibrous drug delivery systems represents a key parameter. Depending on the route of administration and the intended therapeutic application, either rapid or prolonged disintegration may be advantageous. It is generally assumed that nanofibers composed of lower-molecular-weight polymers disintegrate more rapidly [9]. At the same time, however, successful electrospinning generally favors polymers of higher molecular weight, as sufficient chain entanglement is required to achieve stable jet formation and continuous fiber production. This interplay highlights a formulation challenge of particular pharmaceutical relevance: polymers that are expected to promote rapid disintegration are not necessarily those that exhibit the most favorable spinnability.

Accordingly, the first objective of this study was to identify a universal viscosity window of PVP solutions that enables the reproducible formation of electrospun fibers with suitable morphology. To achieve this, the complete standard portfolio of commercially available pharmaceutical-grade PVPs (five grades with different molecular weights) was systematically investigated over a broad concentration range. Particular emphasis is placed on identifying the viscosity conditions necessary for the formation of continuous, bead-free fibers, with special attention to low-molecular-weight PVPs (e.g., K-12), for which successful carrier-free fiber formation is not always readily achieved and may require a marked increase in polymer concentration to ensure adequate chain entanglement.

In addition, the further objective was to compare the in vitro disintegration and dissolution behavior of nanofibrous formulations prepared from the different PVP grades. Although lower-molecular-weight polymers are generally expected to yield faster-disintegrating fibers, the high aqueous solubility of PVP suggests that all formulations could dissolve rapidly once in contact with water. Therefore, both in vitro disintegration and dissolution tests were performed to enable a comparative evaluation and to determine whether significant differences could be observed between formulations prepared from different PVP grades.

Importantly, this research is designed as a foundational pre-formulation study. By systematically evaluating pure, drug-free polymer solutions, this study aims to provide a robust, quantitative baseline framework—a ‘spinnability blueprint’—that other formulators can utilize to easily adapt these physical parameters to their own equipment and specific drugs.

## 2. Materials and Methods

### 2.1. Materials

All PVP used (Table 1) was purchased from International Specialty Products, Inc. (ISP; Wayne, NJ, USA) and the ethanol (96% purity) was bought from Thermo Fisher Scientific Inc. (Budapest, Hungary). Phosphate-buffer solution (PBS; pH 7.4) was used as a medium for the in vitro disintegration and dissolution studies and was prepared as follows. 8.00 g sodium chloride, 0.20 g potassium chloride, 1.44 g disodium hydrogen phosphate, and 0.12 g potassium dihydrogen phosphate (all purchased from Molar Chemicals Ltd., Halásztelek, Hungary) were dissolved in 1 L distilled water, and the pH was adjusted to 7.4 by the addition of phosphoric acid.

### 2.2. Preparation of the Polymer Solutions

The microfibers were produced using solution-based single-needle electrospinning. Prior to electrospinning, polymer solutions were prepared by dissolving accurately weighed amounts of PVP in ethanol to obtain the desired weight-to-weight concentrations. The solvent was selected due to its superior physical properties for electrospinning. Its relatively low boiling point (~78 °C) allows for rapid solvent evaporation during the flight of the jet, preventing fiber fusion and ensuring the formation of dry, well-defined fibers [10]. Furthermore, ethanol is an ICH Class 3 (low toxicity) solvent, and previous gas chromatography (GC) studies on PVP-based electrospun systems have confirmed that residual solvent levels routinely fall well below the strict pharmacopeial limits (<5000 ppm) [11]. Additionally, ethanol is an excellent solvent for various poorly water-soluble active pharmaceutical ingredients (e.g., ibuprofen, curcumin, indomethacin, ciprofloxacin), making this solvent system highly advantageous for future drug delivery applications.

The investigation was initiated using the lowest-molecular-weight PVP grade (K-12) and subsequently continued towards higher molecular weights. After sample collection, the morphology was evaluated by scanning electron microscopy (SEM). The next prepared concentrations were selected based on these observations: the polymer concentration was gradually decreased until suitable fiber morphology was achieved. After the optimization of each polymer grade, the study proceeded to the next polymer grade.

Based on this approach, the following concentrations were prepared for the electrospinning process:

- 70, 65, and 60 *w/w*% for Plasdone K-12;
- 60, 50, and 40 *w/w*% for Plasdone C-17;
- 50, 40, and 30 *w/w*% for Plasdone K-25;
- 40, 30, 20, and 10 *w/w*% for Plasdone K-29/32;
- and 15, 10, and 5 *w/w*% for Plasdone K-90.

### 2.3. Determination of the Solution Viscosity

The viscosity of the polymer solutions was measured by IKA Rotavisc me-vi viscosimeter (IKA-Werke GmbH & Co., Staufen, Germany). The measurements were performed using an SP-11 spindle with a plate diameter of 14.6 mm and an immersion depth of 49.2 mm. The tests were performed at 72 rpm for 30 s at room temperature.

### 2.4. Electrospinning of the PVP Solutions

For microfiber preparation, the Spincube electrospinning device (Spinsplit LLC., Budapest, Hungary) was used. The polymer solution was filled into a 5 mL syringe, which was fixed into the Spincube pump. The used 22 G (0.7 × 13 mm) needle was placed at 15 cm from the plate collector. The feeding rate was set between 0.3 and 1.0 mL/h, while the voltage ranged between 10 and 20 kV. To ensure the reliability and reproducibility of the process, multiple independent production batches were prepared using identical PVP concentrations and electrospinning settings. Special attention was given to formulations located at the boundaries of the spinnability viscosity window. These samples were produced in more independent replicates to verify the consistency of their morphology and fiber diameters. Furthermore, the fibrous mats subjected to in vitro testing were also sourced from these different independent batches.

The preparation was performed under ambient laboratory conditions. Ambient temperature and relative humidity were continuously monitored and recorded during the processes. As the setup did not include an active climate-control chamber, supplementary control trials were conducted across the naturally occurring environmental variations. These preliminary validation trials confirmed that the encountered fluctuations in temperature and humidity did not alter the spinnability or the morphology, further underscoring the robustness of the established process parameters.

### 2.5. Investigation of the Morphology

The morphology of the prepared samples was examined by SEM (Hitachi S4700, Hitachi Scientific Ltd., Tokyo, Japan). The samples were coated with a thin layer of gold–palladium coating (Bio-Rad Sputter Coater 502, VG Microtech, Uckfield, UK) before capturing. The average diameter of the fibers and particles was determined from the SEM images using ImageJ 1.53k software (U. S. National Institutes of Health, Bethesda, MD, USA) based on a total of 100 individual measurements from 3 to 5 independent SEM images taken from different macroscopic regions of the electrospun mats.

### 2.6. Physicochemical Characterization

The physicochemical characterization of the prepared nanofibrous membranes was carried out using two methods. Thermogravimetric analysis was performed simultaneously with heat flow measurement (TGA–DSC) to determine the residual solvent content, while optical contact angle (OCA) measurements were performed to evaluate the wetting behavior.

Thermoanalytical measurements were carried out using approximately 3 mg of each sample, which was placed in an aluminum pan and analyzed using a METTLER-Toledo TGA/DSC 1 thermogravimetric analyzer (Mettler-Toledo GmbH, Gießen, Germany). The measurements were carried out in the temperature range of 25 to 200 °C, with a 5 °C/min heating rate, and under a nitrogen atmosphere with a constant flow rate to prevent oxidative degradation. The results were evaluated using the STARe software version 16.30.

The wetting behavior was evaluated using a DataPhysics OCA20 optical contact angle analyzer (DataPhysics Instruments GmbH, Filderstadt, Germany) employing the sessile drop method. Polar and nonpolar probe liquids (water and diiodomethane, respectively) were deposited onto the surface of the fibrous membranes. The droplet formation and spreading process were recorded and subsequently analyzed using DataPhysics SCA20 software.

### 2.7. In Vitro Disintegration and Dissolution Tests

The disintegration time and the dissolution time of the microfibrous samples were investigated by the same “free immersion” method, which is commonly applied for the evaluation of rapidly disintegrating formulations [12,13,14]. First, ~3 mg of a square specimen was cut out of the fibrous mat and weighed accurately. The specimen was then immersed in 40 mL of the medium while being recorded. PBS pH 7.4 was selected as the standard medium due to its physiological relevance (e.g., blood, extracellular fluid, and saliva). In the case of the disintegration time, the endpoint was the moment when no coherent pieces were visible on the video, while in the case of dissolution time, the endpoint was complete dissolution, i.e., a transparent fluid in the Petri dish. The test was performed three times for each sample, and the average and standard deviation were reported.

### 2.8. Statistical Analysis

Statistical analysis was carried out to determine the existence of a significant difference between the measured data. A one-way Analysis of Variance (ANOVA) with Tukey HSD Test was performed on the fiber diameter and the in vitro disintegration and dissolution data. The results with *p* < 0.01 were assumed to be statistically significant.

## 3. Results

### 3.1. Systematic Evaluation of PVP Molecular Weights and Concentrations in Electrospun Fiber Formation

As discussed above, the fabrication of nano- and microfibers by electrospinning is governed by several parameters, among which the viscosity of the polymer solution and the applied voltage are of primary importance.

The aim of this study was to evaluate the electrospinnability of PVP solutions of different molecular weights and concentrations in order to define a viscosity window that enables stable fiber formation with suitable morphology.

#### 3.1.1. Electrospinning of PVP K-12

First, the lowest-molecular-weight PVP, Plasdone K-12, was electrospun in three different concentrations, namely 70, 65, and 60 *w/w*% (Table 2). The applied voltage and the feeding rate were maintained constant by the electrospinning device. Also, the ambient conditions, including chamber temperature and relative humidity, were considered stable throughout the experiments.

**Figure 2:**
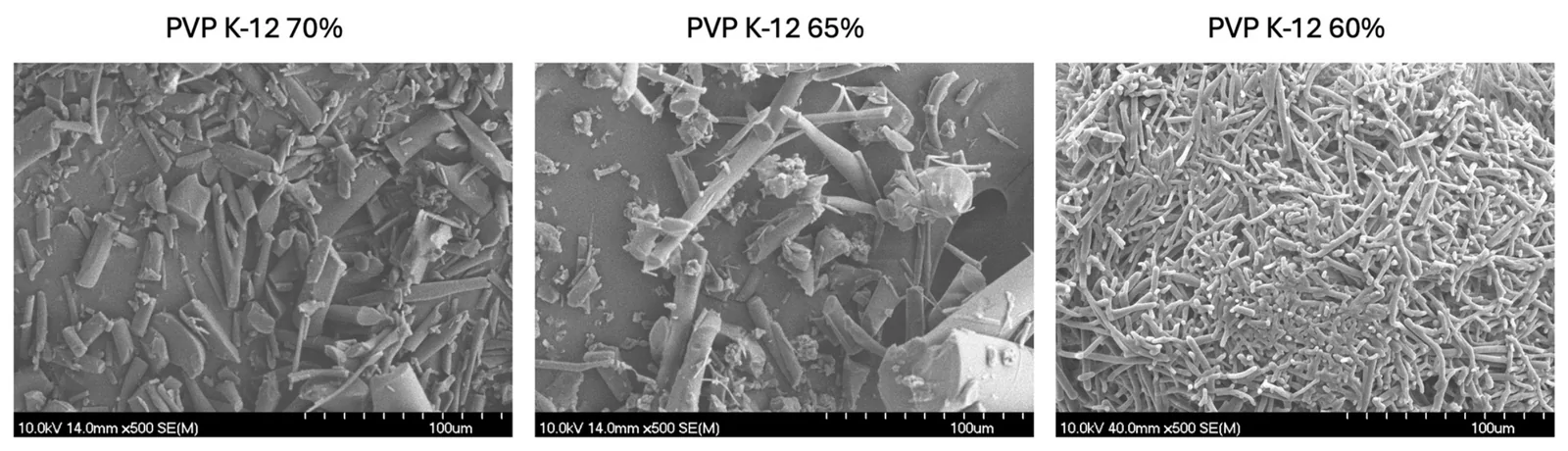
SEM images of the obtained microparticles by electrospinning of PVP K-12 solutions in different concentrations. Microfiber preparation was successful only from the 60 *w/w*% PVP solution.

According to the results, increasing the PVP concentration from 60 to 70 *w/w*% resulted in more than an eightfold increase in solution viscosity. Consequently, the investigated viscosities covered a broad range from 235 to 2043 mPa·s. Electrospinning was attempted for all formulations; however, fibrous structures were successfully obtained only from the PVP K12 60% sample. The morphology of the prepared PVP microparticles is presented in Figure 2.

For the 70% and 65% PVP solutions, the viscosity was too high. In these cases, the applied voltage of 13 kV, which was sufficient under other conditions, as well as charge accumulation on the jet, were not adequate to induce proper jet stretching. As a result, stable fiber formation could not be achieved. Short, thick fragments were collected with diameters of 10.09 ± 5.07 µm and 6.23 ± 3.26 µm in the cases of PVP K-12 70% and 65%, respectively. Previous studies have shown that excessively high solution viscosity restricts jet stretching, leading to instability, nozzle clogging, and the formation of fragmented or ribbon-like structures rather than continuous fibers [2,5].

In contrast, the viscosity of the 60% PVP solution was suitable for microfiber formation. Although continuous fibers were obtained, their relatively short length may be attributed to the low molecular weight of the polymer. The average fiber diameter was 1.98 ± 0.32 µm.

#### 3.1.2. Electrospinning of PVP C-17

The second PVP grade in ascending order of molecular weight was Plasdone C-17. Electrospinning was attempted using solutions with concentrations of 60, 50, and 40 *w/w*% (Table 3). During the experiments, the applied voltage, solution feed rate, and chamber temperature were kept constant. Although variations in relative humidity were observed, they did not have a significant influence on the electrospinning outcome.

**Figure 3:**
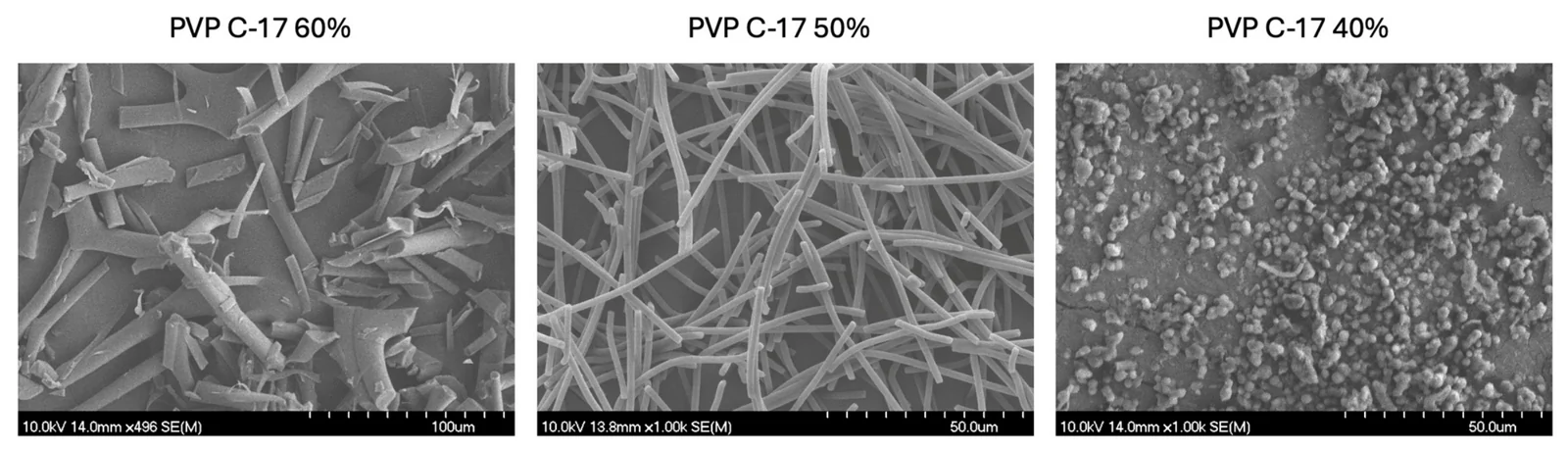
SEM images of the obtained microparticles by electrospinning of PVP C-17 solutions in different concentrations. Microfiber preparation was successful only from the 50 *w/w*% PVP solution.

Prior to electrospinning, the viscosities of the PVP solutions were measured (Table 3). Following electrospinning, the morphology of the collected microstructures was examined (Figure 3). The results were compared with those obtained for the PVP K-12 formulations.

The viscosity of the C-17 60% sample was comparable to that of the PVP K-12 70% and 65% solutions. Accordingly, electrospinning resulted in the formation of thick, irregular fragments with an average diameter of 7.37 ± 2.64 µm rather than continuous fibers. In contrast, the viscosity of the C-17 50% PVP solution (427 mPa·s) proved suitable for fiber fabrication, yielding smooth-surfaced microfibers with an average diameter of 1.74 ± 0.09 µm. At a PVP concentration of 40 *w/w*%, the solution viscosity decreased markedly to 47 mPa·s, which was insufficient for fiber formation. Therefore, electrospraying occurred, resulting in the formation of spherical microparticles with an average diameter of 2.48 ± 0.29 µm.

#### 3.1.3. Electrospinning of PVP K-25

The next PVP grade investigated had a molecular weight of 34,000 (Plasdone K-25). Polymer solutions with concentrations of 50, 40, and 30 *w/w*% were prepared and subjected to electrospinning (Table 4). The viscosity values of the solutions and the morphology of the resulting solid structures were evaluated and systematically compared with the results obtained for the previously investigated PVP grades.

**Figure 4:**
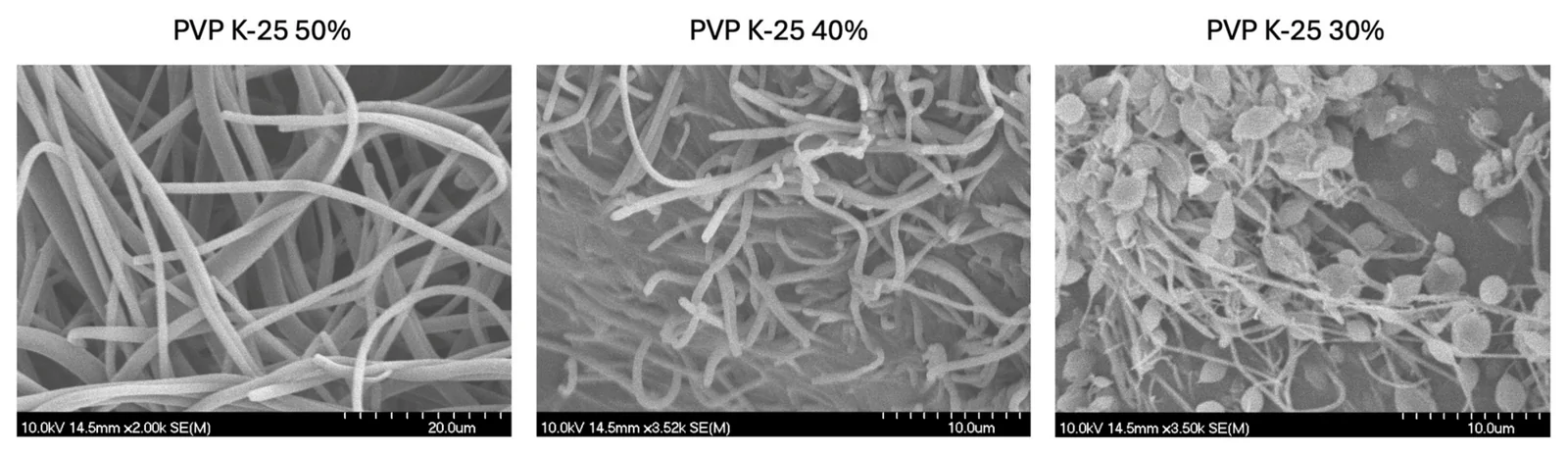
SEM images of the obtained microparticles by electrospinning of PVP K-25 solutions in different concentrations. Microfiber preparation was successful from the 50 and 40 *w/w*% PVP solutions.

Although the viscosity of the PVP K-25 solution at 50 *w/w*% (679 mPa·s) was only slightly lower than that of the PVP K-12 solution at 65 *w/w*% (691 mPa·s), electrospinning was successful in this case (Figure 4). This difference in electrospinnability can most likely be attributed to the higher molecular weight of the polymer. Polymer chains with higher molecular weight facilitate the formation of a stable and continuous jet, which is a prerequisite for proper fiber formation. Accordingly, the more than eightfold increase in molecular weight was sufficient to enable microfiber formation despite the comparable viscosity values. The resulting fibers exhibited suitable morphology with a smooth surface and an average fiber diameter of 1.76 ± 0.63 µm.

Furthermore, a PVP K-25 concentration of 40 *w/w*%, corresponding to a viscosity of 150 mPa·s, was also adequate for electrospinning. Under these conditions, defect-free nanofibers with uniform morphology and thickness were obtained. The average fiber diameter decreased to approximately one-third of that observed for the 50 *w/w*% K-25 formulation, yielding the thinnest nanofibers produced in this study, with a mean diameter of 578 ± 93 nm.

The viscosity of the K-25 solution at 30 *w/w*% (43 mPa·s) was comparable to that of the PVP C-17 solution at 40 *w/w*% (47 mPa·s), and electrospinning was not successful under these conditions. The collected solid structures exhibited a distinctive morphology, characterized by partial fiber formation appearing as fibrous protrusions extending from larger polymer microspheres. The average diameters of the polymer spheres and the fibrous projections were 2.42 ± 0.50 µm and 0.39 ± 0.10 µm, respectively.

#### 3.1.4. Electrospinning of PVP K-29/32

As the next step, the electrospinnability of PVP K-29/32 solution was tested in four different concentrations, namely 40, 30, 20, and 10 *w/w*% (Table 5).

The viscosity of the K-29/32 solution at a concentration of 40 *w/w*% was between the PVP K-25 solutions at 50 and 40 *w/w*%, suggesting that fiber formation would be feasible. Accordingly, electrospinning under these conditions resulted in the formation of smooth-surfaced, continuous microfibers with an average fiber diameter of 1.26 ± 0.39 µm (Figure 4).

In all the other cases, the solution viscosity was insufficient to support stable fiber formation. Consequently, electrospraying occurred for the 20 and 10 *w/w*% PVP K-29/32 solutions, yielding polymer microspheres. Notably, the lower polymer concentration resulted in smaller, smooth-surfaced, and nearly perfectly spherical particles. In contrast, the morphology of the PVP K-29/32 20 *w/w*% sample closely resembled that of the PVP C-17 40 *w/w*% formulation, with both systems producing particles characterized by rough surfaces and irregular shapes (Figure 2 and Figure 4). The measured average particle diameters were 1.57 ± 0.37 µm and 2.14 ± 0.58 µm, respectively.

**Figure 5:**
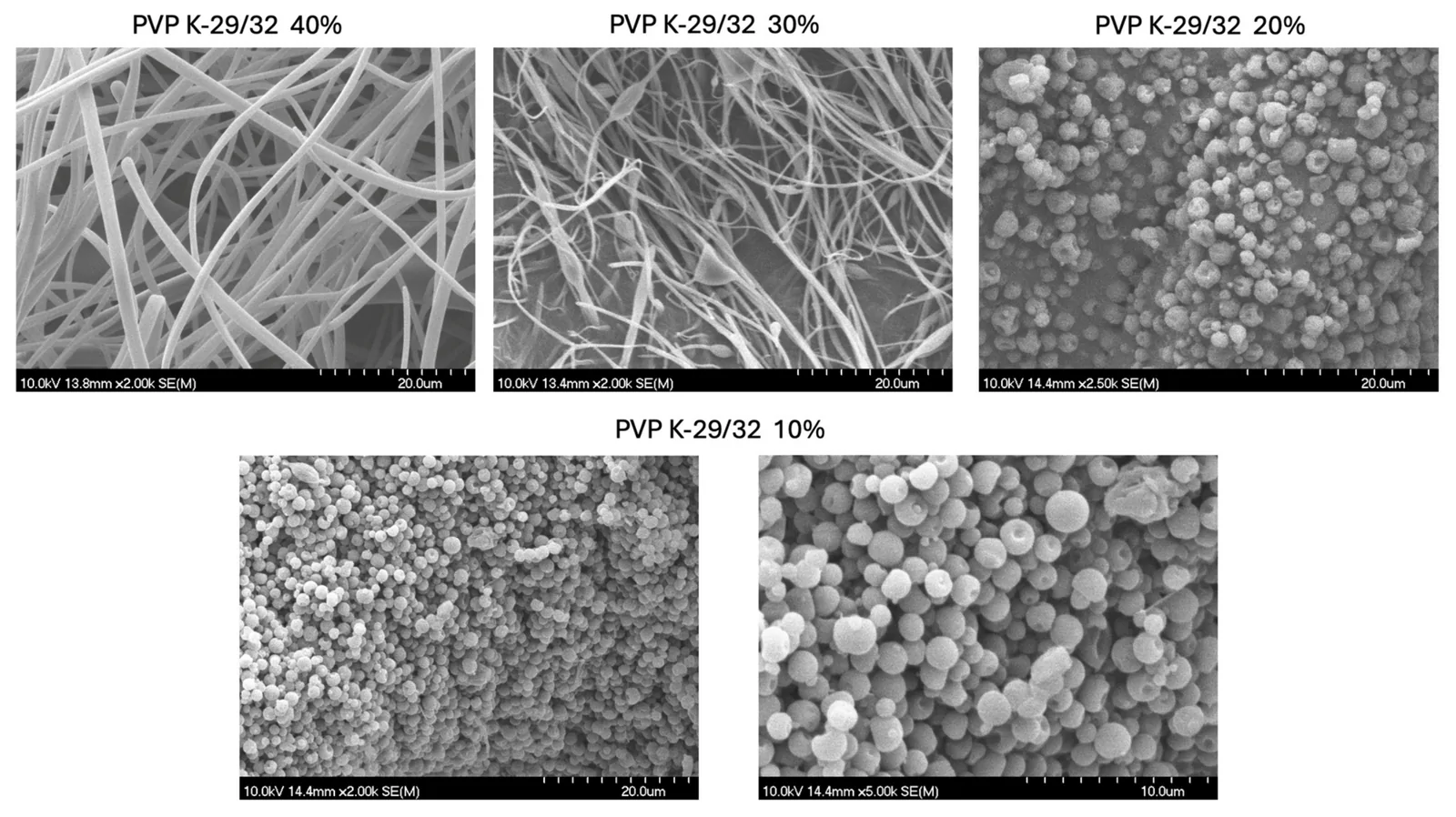
SEM images of the obtained microparticles by electrospinning of PVP K-29/32 solutions in different concentrations. Microfiber preparation was successful only from the 40 *w/w*% PVP solution.

The morphology of the fibers obtained from the PVP K-29/32 30 *w/w*% solution indicated that the solution viscosity (114 mPa·s) was slightly below the lower threshold of the optimal PVP viscosity window (Figure 5). Although fiber formation was initiated, a high number of large beads were observed along the fibers, clearly indicating insufficient viscosity. Owing to the pronounced bead formation and the resulting non-uniform morphology, this formulation was not considered suitable for the in vitro disintegration and dissolution studies.

#### 3.1.5. Electrospinning of PVP K-90

Finally, the PVP grade with the highest molecular weight, Povidone K-90, was subjected to electrospinning at polymer concentrations of 15, 10, and 5 *w/w*% (Table 6).

**Figure 6:**
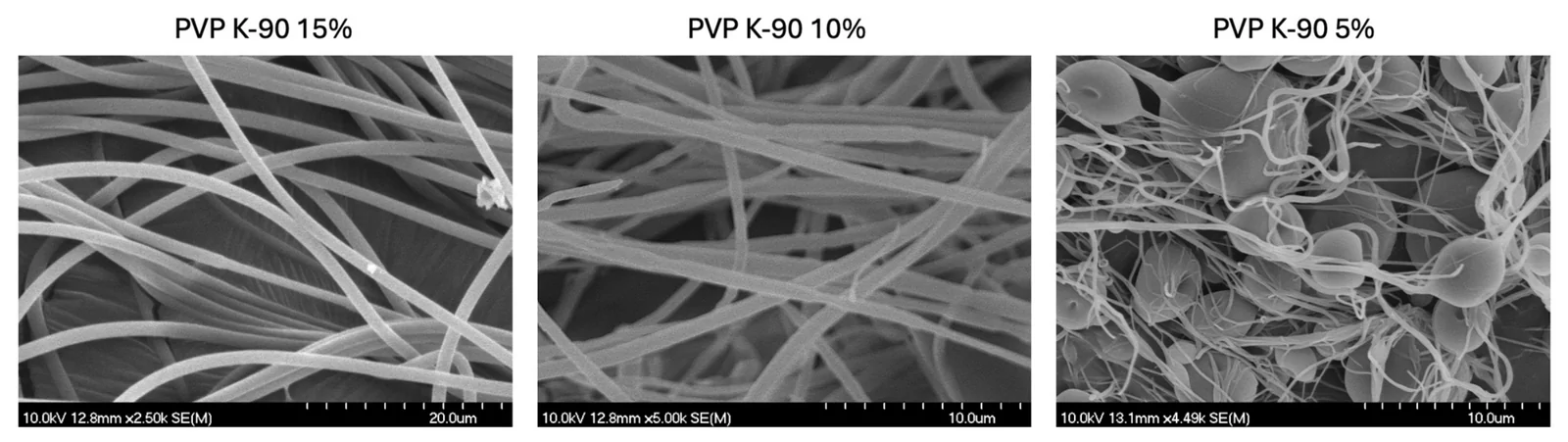
SEM images of the obtained microparticles by electrospinning of PVP K-90 solutions in different concentrations. Microfiber preparation was successful from the 15 and 10 *w/w*% PVP solutions.

Both the solution viscosity and the resulting morphology of the PVP K-90 series closely resembled those observed for the PVP K-25 formulations. Specifically, the viscosity of the PVP K-90 solution at 15 *w/w*% (657 mPa·s) was comparable to that of the PVP K-25 solution at 50 *w/w*% (679 mPa·s). Similarly, the viscosity of the PVP K-90 solution at 10 *w/w*% (164 mPa·s) was close to that measured for the PVP K-25 solution at 40 *w/w*% (150 mPa·s). As both K-25 formulations at 50 and 40 *w/w*% concentrations resulted in successful fiber formation, the production of uniform, smooth-surfaced fibers was also anticipated for the corresponding K-90 solutions (Figure 6).

Accordingly, electrospinning of the higher-concentration K-90 solution yielded continuous microfibers, whereas the lower polymer concentration resulted in the formation of nanofibers. The average fiber diameters were 1.56 ± 0.27 µm and 886 ± 217 nm for the microfibrous and nanofibrous samples, respectively. In contrast, the morphology of the PVP K-90 solution at 5 *w/w*% was characterized by fibrous protrusions extending from larger polymer microspheres, similar to the structures observed for the PVP K-25 solution at 30 *w/w*%. In this case, the solution viscosity (21 mPa·s) was insufficient to sustain stable electrospinning, further confirming that a minimum viscosity threshold is required for proper fiber formation.

### 3.2. Defining the Viscosity Window for Stable Electrospinning

Based on the obtained results, it was clearly demonstrated that the viscosity of the polymer solutions plays a decisive role in fiber formation. PVP grades with different molecular weights require distinct optimal concentration ranges to achieve successful nanofiber fabrication. An appropriate viscosity window ensures stable, continuous, and homogeneous jet formation during electrospinning, whereas excessively low viscosity leads to electrospraying and particle formation, while excessively high viscosity results in discontinuous jets and fragmented structures.

**Figure 7:**
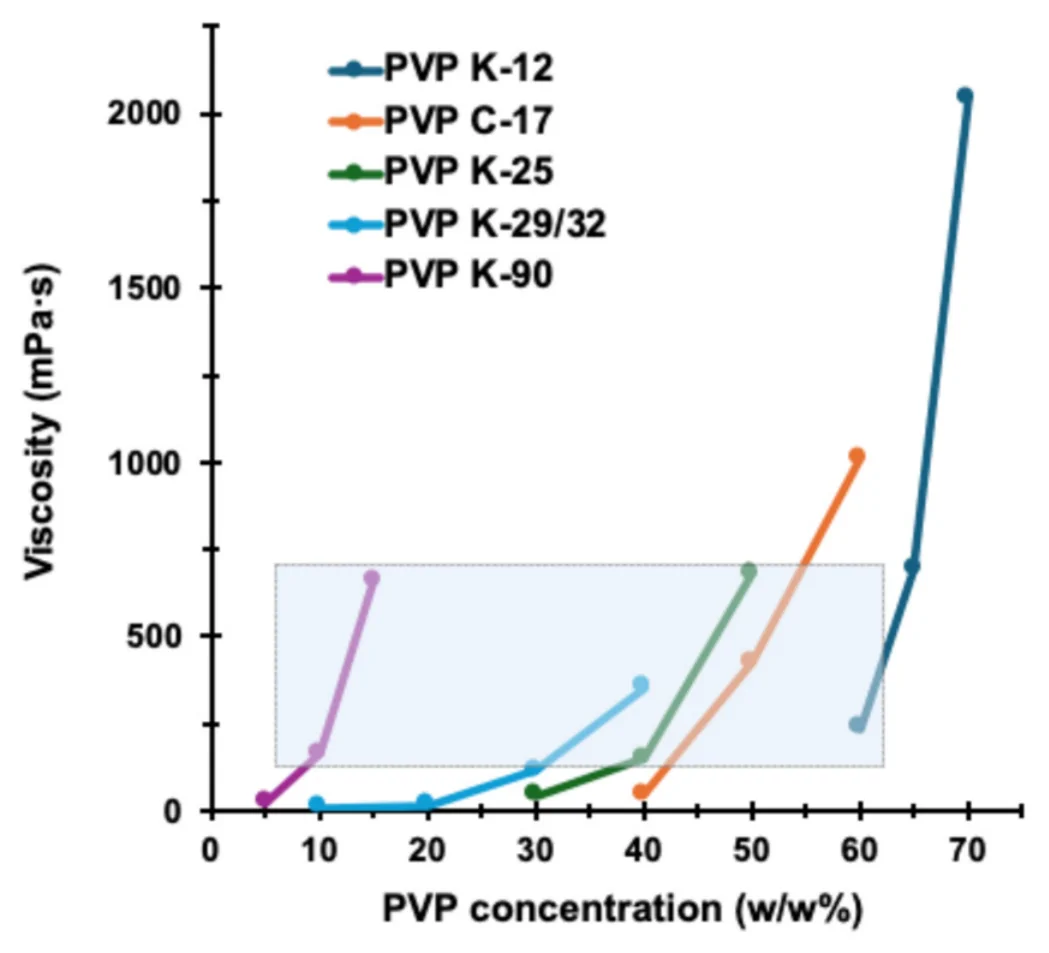
Viscosity values as a function of PVP concentration and polymer grade. The blue shaded region represents the optimal viscosity window, within which stable and continuous fiber formation was achieved during the electrospinning.

On this basis, an optimal viscosity window for reliable nanofiber formation can be defined. The results indicate that PVP concentrations in the range of 10–60 *w/w*% and solution viscosities of approximately 150–680 mPa·s are favorable for the formation of continuous and uniform PVP nanofibers under the applied electrospinning conditions (Figure 7).

Table 7 summarizes the samples that successfully yielded nanofibers, arranged in ascending order of solution viscosity. It is worth emphasizing that electrospinning was also feasible in the case of low-molecular-weight PVP, provided that solutions of sufficiently high concentration were prepared. It is important to note that the entanglement concentration (Ce) plays a key role in the formation of a stable polymer jet and, consequently, in successful electrospinning. It was observed that the high-molecular-weight PVP K-90 sample at 15 *w/w*% exhibited a viscosity comparable to that of the significantly lower-molecular-weight PVP K-25 at 50 *w/w*%. Since chain entanglement is a critical factor in the electrospinning process, polymers with lower molecular weight must be used at substantially higher concentrations to achieve adequate chain entanglement and fiber formation.

In general, higher solution viscosity is associated with increased fiber diameter [5,15]. This trend was also clearly observed in the present study, as PVP solutions with viscosities of approximately 150–160 mPa·s yielded nanometer-scale fibers, whereas more viscous PVP solutions resulted in microfibers. As the fiber morphology obtained from the PVP K-12 60% sample resulted in not optimal, short and thick fibers, this sample was excluded from the general observations.

One-way ANOVA demonstrated that solution viscosity had a statistically significant effect on fiber diameter (*p* < 0.01). Subsequent post hoc analysis confirmed the presence of three distinct groups with significantly different average fiber diameters.

The first group comprised low-viscosity solutions producing submicron fibers. The second group included solutions with viscosities in the range of 430–680 mPa·s, which generated fibers with diameters exceeding 1.5 µm. The third group included a sample with intermediate viscosity (354 mPa·s). This formulation exhibited fiber diameters that differed significantly from those observed in both the lower- and higher-viscosity groups.

### 3.3. Thermal Behavior and Liquid Uptake Characteristics

Since residual solvent and moisture absorbed from the environment can act as plasticizers, thermoanalytical characterization of the samples was considered essential. Therefore, fibrous samples with appropriate morphology were subjected to both TGA and DSC measurements to evaluate the presence of bound water and residual ethanol.

**Figure 8:**
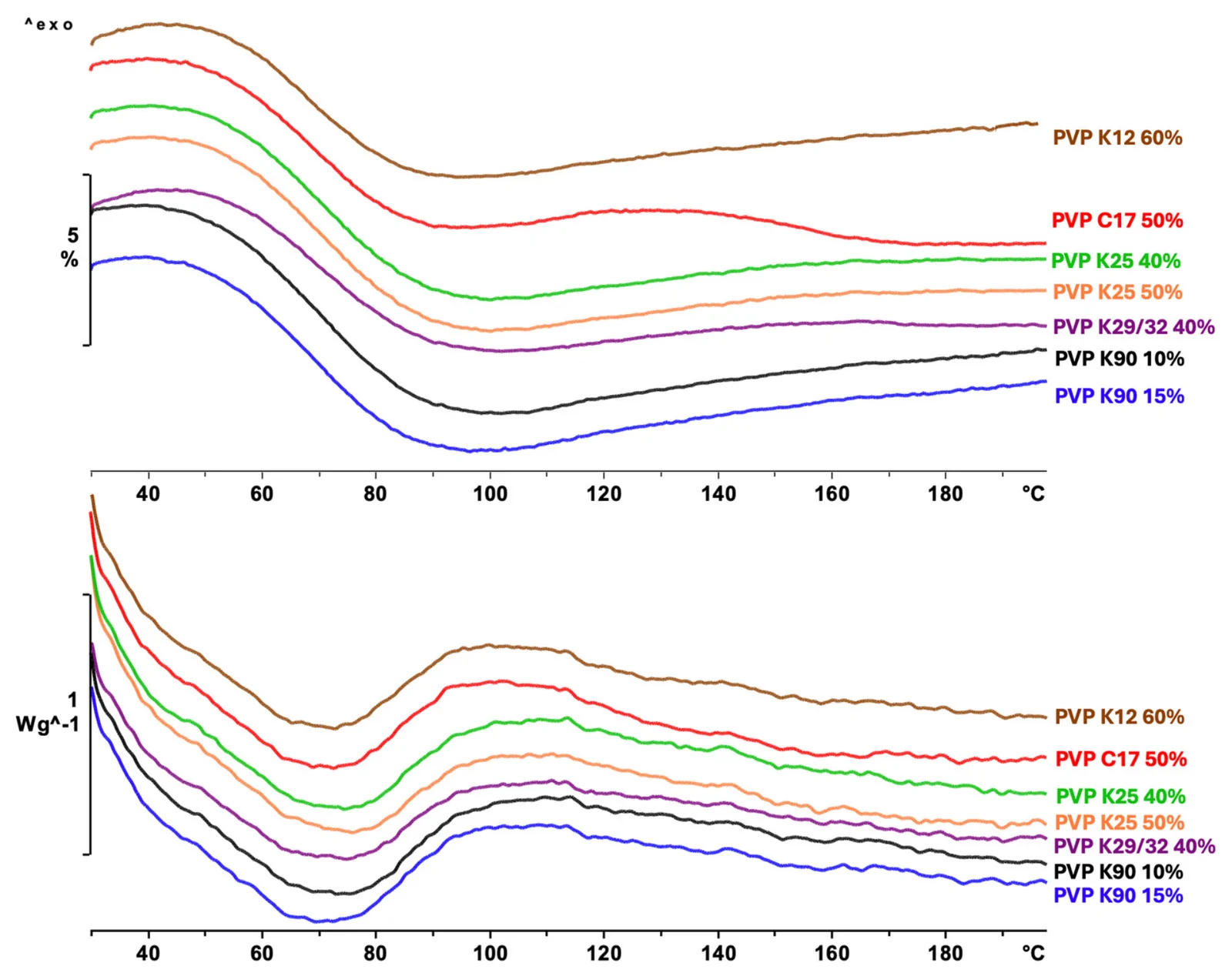
Thermal analysis of the electrospun PVP fibers with varying molecular weights. The top panel shows the TGA curves, where the vertical scale bar indicates a 5% weight loss. The bottom panel displays the DSC heating curves, where the vertical scale bar indicates a heat flow of 1 W/g.

In all cases, a similar temperature-dependent behavior was observed, regardless of the molecular weight of PVP (Figure 8). The consistent shape of the curves indicates that the same thermal processes occur across all formulations. The extent of mass loss (TGA) and the intensity of the corresponding endothermic peaks (DSC) do not exhibit a monotonic variation among the samples; the minor differences reflect normal sample-to-sample variability rather than a molecular weight-driven trend.

As Figure 8 shows, pronounced mass loss occurs in the temperature range of approximately 50–90 °C, accompanied by a broad endothermic peak in the DSC heat flow curves. This coupled thermal event clearly indicates the desorption of bound water from the fibrous samples. Notably, no separate thermal event assignable to the evaporation of ethanol was observed. While this confirms that ethanol is not present in significant quantities, the existence of trace amounts overlapping with the broad water desorption band cannot be entirely excluded.

**Figure 9:**
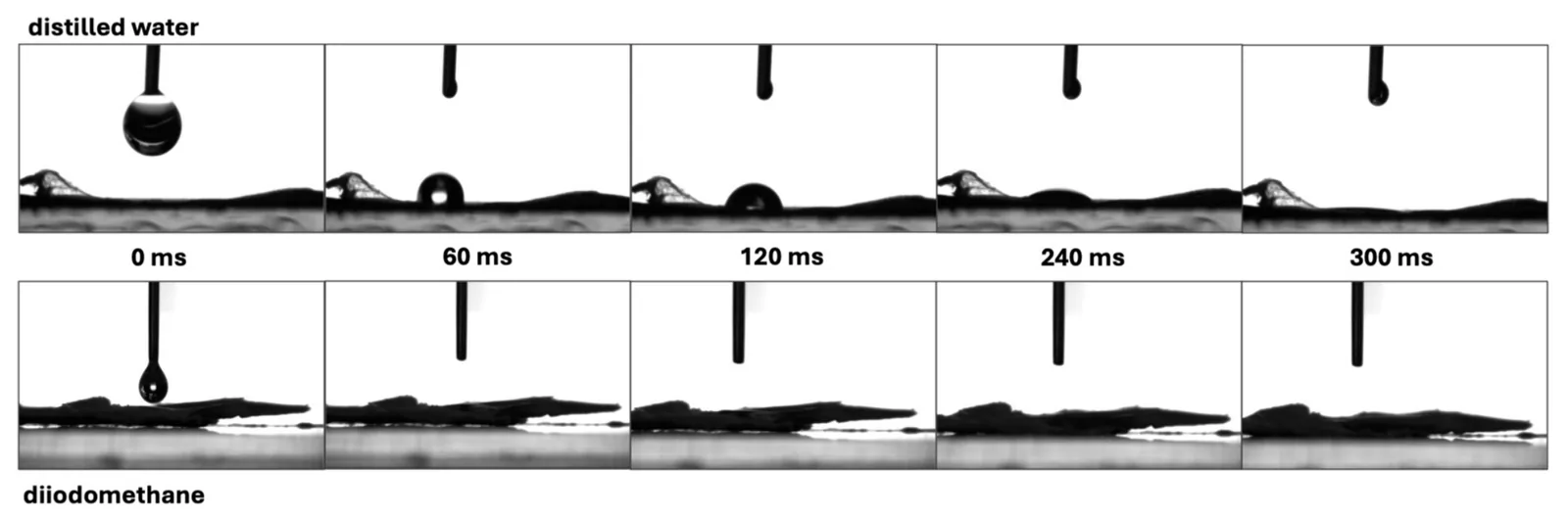
Series of images illustrating the absorption of water and diiodomethane on fibrous PVP membranes.

Contact angle measurements of water-soluble materials present significant challenges, as the liquid may dissolve or rapidly penetrate the sample. In the case of highly porous fibrous structures, liquid uptake is dominated by capillary forces rather than surface wetting, preventing the formation of a stable droplet. In this study, both distilled water and diiodomethane were dispensed onto the surface of the fibrous membranes to test the wettability and contact angle. However, due to the water-soluble and porous nature of the samples, rapid liquid absorption occurred in both cases, making conventional contact angle determination unreliable. Figure 9 shows that absorption occurred immediately, within less than 300 ms for distilled water and approximately 60 ms for diiodomethane. This significant time difference is attributed to two main factors. First, due to its high surface tension (72.8 mN/m), water initially traps air within the pores (forming a transient Cassie–Baxter state). It requires a brief time to displace this air and transition into a fully wetted Wenzel state [16]. Second, water instantly hydrates and swells the PVP fibers, forming a viscous gel layer that partially constricts the capillary channels and slows imbibition. In contrast, diiodomethane has a lower surface tension (50.8 mN/m) and does not swell PVP, allowing for immediate pore penetration, open capillary channels, and unhindered, significantly faster wicking. Ultimately, the fibrous structure enabled the rapid penetration of both polar and nonpolar liquids. These ultrafast absorptions indicate that liquid uptake is dominated by capillary forces rather than surface wetting.

### 3.4. Comparison of In Vitro Disintegration and Dissolution Times

**Figure 10:**
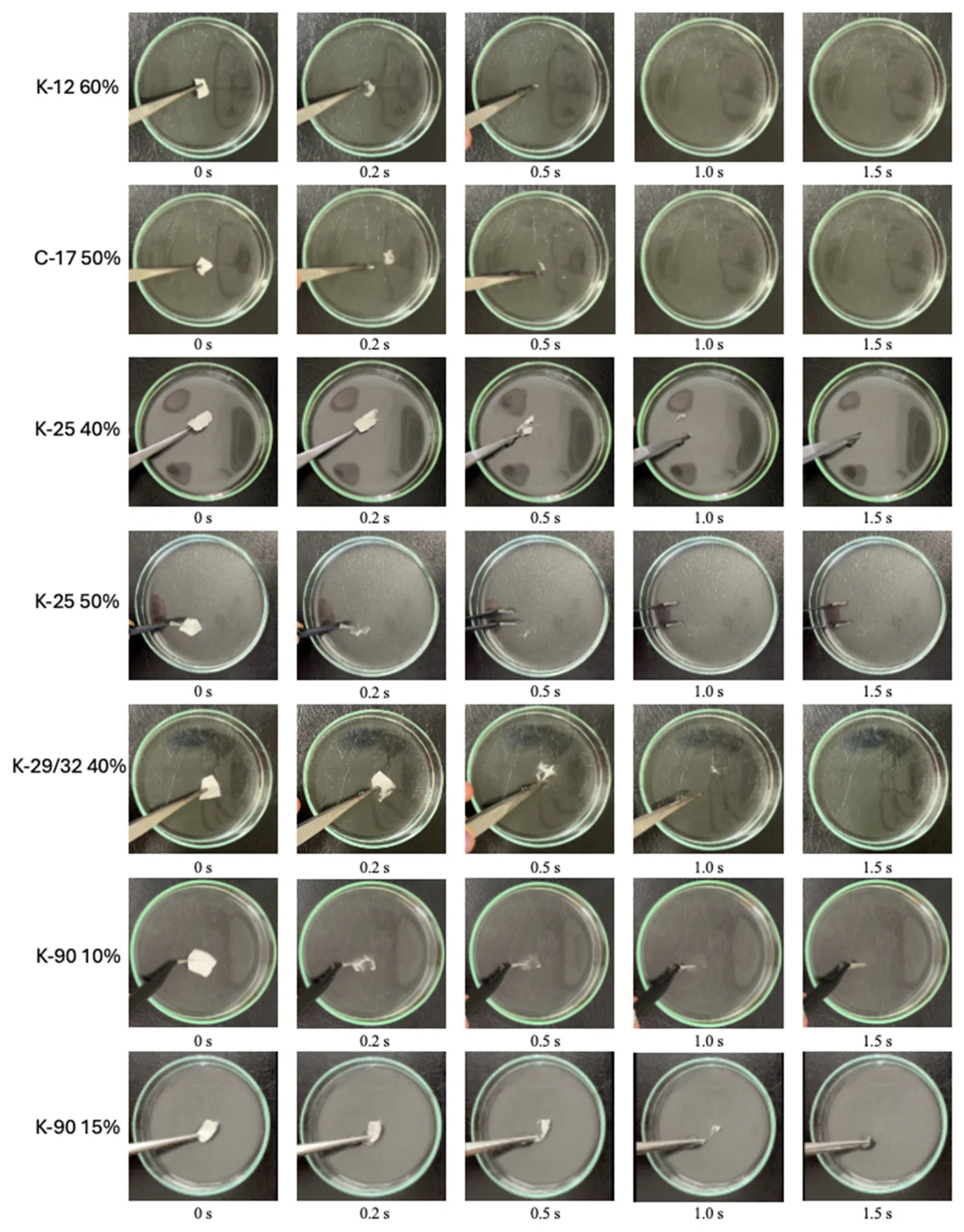
Series of images illustrating the disintegration and dissolution times of the PVP-based fibrous samples.

The in vitro disintegration and dissolution behavior of the seven fibrous formulations listed in Table 7 was further investigated using a free immersion method to evaluate whether the molecular weight of PVP or the fiber diameter influenced these parameters. The obtained results are summarized in Table 8 and illustrated in Figure 10.

The results demonstrate that the fibrous mats disintegrated within 0.29–1.33 s, while PVP dissolution occurred within 0.39–2.32 s, independent of both polymer molecular weight and fiber diameter. A one-way ANOVA revealed no statistically significant differences in the disintegration times (F(6,14) = 1.66; *p* = 0.202) and dissolution times (F(6,14) = 1.84; *p* = 0.162) across the different formulations. These statistical findings suggest that the ultrafast disintegration and dissolution kinetics are not limited by the molecular weight of the specific PVP grade. Instead, the process is entirely dominated by the macroscopic structural features of the electrospun webs—namely, the high porosity and the resulting capillary-driven fluid uptake.

## 4. Discussion

Although solution viscosity is widely recognized as a critical parameter in electrospinning, it remains insufficiently reported in the literature. Therefore, in this study, particular emphasis was placed on defining a viscosity window enabling the reproducible formation of continuous, bead-free PVP fibers across a wide molecular weight range. Under the applied conditions, this optimal range was identified as approximately 150–680 mPa·s. Within this window, polymer solutions exhibit a balance between sufficient chain entanglement and adequate processability, both of which are essential for maintaining a stable and continuous jet during electrospinning.

This viscosity range can be interpreted in terms of the transition between concentration regimes. The lower boundary corresponds to the onset of sufficient chain overlap above the Ce, where an interconnected polymer network is formed, providing the viscoelastic stability required for jet elongation. In contrast, the upper boundary represents a regime in which excessively high viscosity limits elongational flow and restricts jet stretching. Although no explicit mathematical model was established, this semi-quantitative framework facilitates defining the optimal processing range which allows comparison with other polymer–solvent systems.

Within this established viscosity regime, PVP nanofibers were successfully fabricated from polymers covering a broad molecular weight interval (Mw 4000–1,300,000). Importantly, existing studies do not describe electrospun nanofibers composed solely of PVP with molecular weights comparable to PVP K-12, primarily due to insufficient chain entanglement required to sustain a continuous electrospinning jet. In the present work, however, continuous nanofibers were successfully obtained from all investigated PVP grades, including the extremely low-molecular-weight PVP K-12. Consequently, the successful formation of continuous nanofibers from this polymer represents a particularly remarkable finding.

This observation highlights that solution viscoelastic properties, governed by both concentration and chain entanglement, play a more dominant role than molecular weight alone in determining electrospinnability, while also providing experimental evidence that the limitations imposed by low chain entanglement can be effectively mitigated. The observed behavior is in good agreement with the concept proposed by Maduna et al., who suggested that higher polymer concentrations can compensate for reduced chain entanglement in low-molecular-weight systems [8]. Consistently, in the present case, continuous fiber formation from PVP K-12 (Mw 4000) was only achieved at a high concentration of 60 wt%, confirming the critical interplay between molecular weight, concentration, and viscoelasticity in determining electrospinnability.

Another key aspect of this study was to assess the influence of PVP molecular weight on disintegration time. Due to its excellent water solubility, PVP-based nanofibrous systems typically exhibit rapid disintegration and drug release (Table 9), although sustained release has also been reported in certain cases [13,17,18]. In such studies, either the disintegration was not explicitly evaluated or occurred over significantly longer times compared to fast-dissolving systems.

Mutlu-Ağardan et al. reported that nanofibers fabricated from lower-molecular-weight PVP K30 exhibit substantially faster drug release than those prepared from higher-molecular-weight PVP K90, suggesting a strong dependence of release kinetics on polymer chain length [17]. Since disintegration is a prerequisite for drug dissolution, understanding the factors influencing this process is essential. Accordingly, the present work aimed to elucidate the role of molecular weight in the disintegration behavior of PVP nanofibers.

The obtained results showed that disintegration (0.29–1.33 s) and dissolution (0.39–2.32 s) occurred rapidly for all samples, independent of polymer molecular weight and fiber diameter, with no statistically significant differences. Thus, these findings contradict the common assumption that higher-molecular-weight PVP would lead to slower disintegration. Instead, the results indicate that molecular weight alone does not govern disintegration kinetics in electrospun systems.

The rapid dissolution can be mechanistically explained by the excellent wettability of PVP, combined with the exceptionally high specific surface area and the capillary-driven liquid penetration of the fibrous structure. These features dominate the disintegration process and effectively overshadow the influence of molecular weight. Therefore, in electrospun PVP systems, structural and morphological factors appear to play a more decisive role in determining disintegration behavior than polymer chain length.

For comparison, other water-soluble polymers commonly used in electrospinning, such as poly(vinyl alcohol) (PVA) and hydroxypropyl methylcellulose (HPMC), have been investigated in fast-dissolving drug delivery systems. As PVA is a highly hydrophilic polymer, PVA nanofibers rapidly disintegrate upon contact with water [24,25]. Topal et al. investigated PVA/PVP blended nanofibers loaded with anti-Alzheimer drugs. The fibers exhibited rapid wetting (<1 s), followed by the onset of disintegration at around 21 s and near-complete dispersion within approximately 110 s in simulated saliva [26]. Similarly, Mortazavi et al. reported that electrospun HPMC-based nanofiber mats exhibited significantly faster disintegration compared to solvent-cast films (3 s vs. 30 s), alongside faster drug release [27]. These findings clearly indicate that the superior performance of electrospun systems can be primarily attributed to their nanofibrous structure, characterized by high specific surface area and porosity, rather than the intrinsic properties of the polymer alone. However, it should be emphasized that this behavior is primarily characteristic of highly water-soluble polymers, where rapid hydration and dissolution enable the nanofibrous structure to dominate disintegration and drug release processes.

It should also be noted that all measurements were conducted at 25 °C under controlled laboratory conditions. At physiological temperature (37 °C), faster hydration, increased polymer chain mobility, and enhanced diffusion might be expected, which could result in even shorter disintegration times. This further supports the practical applicability of the developed systems for fast-dissolving formulations.

## 5. Conclusions

This study successfully established a comprehensive pre-formulation blueprint by systematically investigating the complete standard portfolio of commercially available pharmaceutical-grade PVPs. A well-defined universal viscosity window for PVP solutions was pinpointed, enabling the reproducible electrospinning of fibrous mats with controlled morphology. Stable and homogeneous nanofiber formation was achieved within a concentration range of 10–60 *w/w*% and a viscosity range of approximately 150–680 mPa·s, whereas deviations from this window resulted in electrospraying at low viscosities and jet instability at high viscosities. Lower-viscosity solutions favored the formation of nanofibers, while higher viscosities led to microfiber production. Notably, operating within this optimized rheological window enabled the successful, carrier-free electrospinning of the exceptionally low-molecular-weight PVP K-12.

Furthermore, the use of ethanol as a single, ICH Class 3 solvent not only ensures process safety but also provides an optimal, easily evaporable foundation for future drug-loading applications.

Independent of polymer molecular weight and fiber diameter, all fibrous formulations exhibited extremely rapid in vitro disintegration and dissolution. The absence of statistically significant differences among samples demonstrates that the molecular weight of PVP does not govern the disintegration kinetics of nano- or microfibrous mats under the investigated conditions. Consequently, the commonly assumed hypothesis that higher-molecular-weight PVP fibers disintegrate more slowly was not supported.

The observed immediate dissolution originates from rapid wetting and capillary-driven liquid uptake within the fibrous network, facilitated by the high wettability of PVP and the exceptionally large specific surface area of the nano- and microfibrous structure.

From a translational perspective, while the exact numerical boundaries of the defined viscosity window will likely require re-optimization for industrial scale-up (e.g., in multi-nozzle or nozzle-free technologies), the fundamental principle of entanglement-driven spinnability and the resulting highly porous, fast-dissolving architecture are expected to be fully preserved.

By establishing this robust, drug-free baseline, these findings highlight the dominant role of structural and physicochemical properties over polymer molecular weight in determining the disintegration performance of PVP-based fibrous systems. Ultimately, this systematic scheme provides a highly practical framework that other researchers and formulators can utilize to easily adapt these physical parameters to their own equipment and specific active ingredients in the development of fast-dissolving drug delivery platforms.

## Figures and Tables

**Table 1:** The attributes of the used polyvinylpyrrolidone (PVP) powders according to the Sales Specifications of the producer.

Brand Name	K-Value	Average Molecular Weight (Mw)	Glass Transition Temperature (°C)
Plasdone K-12	10.2–13.8	4000	120
Plasdone C-17	15.5–17.5	10,000	126
Plasdone K-25	24–26	34,000	160
Plasdone K-29/32	29–32	58,000	164
Plasdone K-90	85–95	1,300,000	174

**Table 2:** The electrospinning parameters and the viscosity values of the PVP K-12 samples.

Sample Name	PVP K-12 70%	PVP K-12 65%	PVP K-12 60%
Polymer concentration (*w/w%*)	70	65	60
Viscosity (mPa·s)	2043	691	235
Voltage (kV)	13	13	13
Feeding rate (mL/h)	0.3	0.3	0.3
Chamber temperature (°C)	22	23	22
Relative humidity (%)	40	41	40

**Table 3:** The electrospinning parameters and the viscosity values of the PVP C-17 samples.

Sample Name	PVP C-17 60%	PVP C-17 50%	PVP C-17 40%
Polymer concentration (*w/w*%)	60	50	40
Viscosity (mPa·s)	1011	427	47
Voltage (kV)	13	13	13
Feeding rate (mL/h)	0.3	0.3	0.3
Chamber temperature (°C)	22	23	24
Relative humidity (%)	40	41	54

**Table 4:** The electrospinning parameters and the viscosity values of the PVP K-25 samples.

Sample Name	PVP K-25 50%	PVP K-25 40%	PVP K-25 30%
Polymer concentration (*w/w*%)	50	40	30
Viscosity (mPa·s)	679	150	43
Voltage (kV)	13	13	13
Feeding rate (mL/h)	0.3	0.3	0.3
Chamber temperature (°C)	23	27	27
Relative humidity (%)	56	51	52

**Table 5:** The electrospinning parameters and the viscosity values of the PVP K-29/32 samples.

Sample Name	PVP K-29/32 40%	PVP K-29/32 30%	PVP K-29/32 20%	PVP K-29/32 10%
Polymer concentration (*w/w*%)	40	30	20	10
Viscosity (mPa·s)	354	114	14	7
Voltage (kV)	10	13	13	15
Feeding rate (mL/h)	0.5	0.8	0.8	0.7
Chamber temperature (°C)	25	23	26	24
Relative humidity (%)	42	33	42	39

**Table 6:** The electrospinning parameters and the viscosity values of the PVP K-90 samples.

Sample Name	PVP K-90 15%	PVP K-90 10%	PVP K-90 5%
Polymer concentration (*w/w*%)	15	10	5
Viscosity (mPa·s)	657	164	21
Voltage (kV)	15	15	10
Feeding rate (mL/h)	1.0	1.0	0.6
Chamber temperature (°C)	25	25	24
Relative humidity (%)	44	39	42

**Table 7:** Average fiber diameter and viscosity values of PVP solutions resulting in continuous fibers.

Sample Name	Viscosity (mPa·s)	Average Fiber Diameter
PVP K-25 40%	150	578 ± 93 nm
PVP K-90 10%	164	886 ± 217 nm
PVP K-12 60%	235	1.98 ± 0.32 µm
PVP K-29/32 40%	354	1.26 ± 0.39 µm
PVP C-17 50%	427	1.74 ± 0.09 µm
PVP K-90 15%	657	1.56 ± 0.27 µm
PVP K-25 50%	679	1.76 ± 0.63 µm

**Table 8:** In vitro disintegration and dissolution time of the continuous fibers.

Sample Name	Average Fiber Diameter (µm)	Disintegration Time (s)	Dissolution Time (s)
PVP K-12 60%	1.98 ± 0.32	0.29 ± 0.08	0.39 ± 0.04
PVP C-17 50%	1.74 ± 0.09	0.94 ± 0.40	1.45 ± 0.37
PVP K-25 40%	0.58 ± 0.09	0.87 ± 0.37	1.50 ± 1.08
PVP K-25 50%	1.76 ± 0.63	0.64 ± 0.31	1.08 ± 0.32
PVP K-29/32 40%	1.26 ± 0.39	1.33 ± 0.98	2.32 ± 1.55
PVP K-90 10%	0.89 ± 0.22	0.47 ± 0.27	1.08 ± 0.23
PVP K-90 15%	1.56 ± 0.27	1.07 ± 0.44	1.89 ± 0.74

**Table 9:** Disintegration and drug release characteristics of PVP-based nanofibers.

PVP Grade	Active Pharmaceutical Ingredient	Electrospinning Parameters	Disintegration Time	Drug Release Time	Ref.
K-29/32	Fenofibrate	15 kV, 0.7 mL/h, 16 G needle, 7.5 cm	instant	1 min	[19]
K-30	Acyclovir	15 kV, 0.5 mL/h, 23 G needle, flat plate, 15 cm, 50% RH, 20 °C	20 s	1 h	[13]
K-30	Silver sulfadiazine-containing nanoparticles	18 kV, 2.5 mL/h, rotating drum (100 rpm), 13 cm	ND	8 h	[17]
K-90	Silver sulfadiazine-containing nanoparticles	16 kV, 2.5 mL/h, rotating drum (100 rpm), 13 cm	ND	24 h	[17]
K-90	Buprenorphine hydrochloride	20 kV, 0.5 mL/h, rotating drum, 15 cm	ND	1,5 h	[18]
K-90	α-Mangostin	10 kV, 5 μL/min, 26 G needle, rotating drum, 12 cm	ND	15 min	[20]
K-90	Lapatinib ditosylate monohydrate	14 kV, 0.9 mL/h, 20 G needle, rotating drum (300 rpm), 10 cm, 30% RH, 20 °C	instant	5 min	[11]
K-90	Diclofenac sodium	15 kV, 0.3–0.8 mL/h, 22 G needle, 25 cm, RT	1.3 ± 0.3 s	5 min	[21]
K-90	Diclofenac sodium + SSG (super-disintegrant)	15 kV, 0.3–0.8 mL/h, 22 G needle, 25 cm, RT	0.4 ± 0.1 s	1 min	[21]
K-90	Diclofenac sodium	16 kV, 0.5 mL/h, double needle (22 G), flat plate, 17 cm, 36–44% RH, 24–25 °C	5.5 ± 1.5 s	4 min	[22]
K-90	Albendazole sulfoxide; Praziquantel	18 kV, 2 mL/h, 20 G needle, rotating drum, 15 cm, RT	<0.8 s	5 min	[23]

## Data Availability

The original contributions presented in this study are included in the article. Further inquiries can be directed to the corresponding author.

## Abbreviations

The following abbreviations are used in this manuscript:

ANOVA	Analysis of variance
Ce	Entanglement concentration
HPMC	Hydroxypropyl methylcellulose
OCA	Optical contact angle
PBS	Phosphate-buffer solution
PVA	Poly(vinyl alcohol)
PVP	Poly(vinylpyrrolidone)
SEM	Scanning electron microscopy
SSG	Sodium starch glycolate
TGA	Thermogravimetric analysis
